# A Case of Injury from Strong Electrical Current

**Published:** 1886-11

**Authors:** Harold N. Moyer

**Affiliations:** Assistant to the Chair for Diseases of the Nervous System and Lecturer on Physiology, Rush Medical College; 434 West Adams Street


THE CHICAGO

MEDICAL JOURNAL AND EXAMINER.

VOL. LIII. NOVEMBER, 1886. No. 5.

### Original Communications.

### A Case of Injury from a Strong Electric Current. *By* Harold N. Moyer, M.D., *Assistant to the Chair for Diseases of the Nervous System and Lecturer on Physiology, Rush Medical College.*

The rarity of injury occasioned by the passage of strong electric currents through the body, and the silence of the standard text-books on surgery regarding this class of injuries, shall be my excuse for presenting what would otherwise seem to be a case of little interest. With the widening of the application of electric currents in the mechanical arts, and electric lighting, must come an increase in the number of these accidents, and a corresponding interest in their surgical and medico-legal relations. I do not remember to have seen a single case reported in which an injury of this kind has been made the subject of a legal inquiry, but, with the increased exposure to violence of this kind, it must soon be recognized, and we shall doubtless shortly have a special department of "electric surgery," similar in many respects to the "railroad surgery" of today.

On the evening of August 5th last I was called to see S. C., a laborer, well developed, muscular, and of a healthy, robust appearance. I found him lying in bed bathed in a cold perspiration. His skin was pale and of a leaden, ashy hue. The pupils were widely dilated and reacted but feebly to light. He was partially delirious, though when pressed to do so he would give fairly rational answers to questions. His pulse was about 100, feeble, and slightly irregular. The heart sounds were normal, though its action was somewhat hurried and irregular. His temperature was 97½° F. Nausea and vomiting were constant; everything taken into the stomach was immediately rejected. On the outer aspect of the knee, about one inch from the patella, was found an oval opening three-fourths of an inch long and one-half an inch wide, extending below the deep fascia. The opening had sharply defined edges which were not everted. The opening showed an actual loss of substance, as though made with a punch. There was very little hæmorrhage at that time, and it was stated that there had been but little. On the posterior aspect of the thigh, in the middle line, just above the popliteal space, a round, button-like eschar, about one-half an inch in diameter, was found. The eschar was of a blackish-brown color, and seemed to involve only the skin, and to be freely movable on the tissues beneath. Careful examination failed to reveal any displacement of the bones or ligaments, but I was unable to assure myself that the synovial membrane of the joint was not involved in the solution of continuity. The possible gravity of this wound of the knee induced me to send the patient to the Cook County Hospital, after having applied a temporary dressing. This course was necessary, from the fact the patient lived in lodgings, and had no one to attend to his wants. At the hospital the wound healed kindly, and in a few days he was again on the street. The eschar on the posterior aspect of the thigh separated, leaving a deep fistulous track extending forwards and downwards, which at the end of five weeks had not yet healed, and long after all other evidence of injury had disappeared.

It was after the patient left the hospital that I obtained full particulars of the manner in which the accident occurred. He was engaged in adjusting one of the arc lights of a thirty-light circuit of the Van De Pœle system. At the time he was standing on a step-ladder a few feet above the sidewalk, his foot slipped, and in attempting to save himself he grasped both of the side wires of the lamp. For an instant at least a portion of the current passed through his body. It is hardly conceivable that a current of this intensity could pass through the body without causing instant death, and I am of the opinion that a portion at least must have still passed through the lamp. In falling his knee came in contact with an iron railing, and the current was grounded. As he expressed it, "the fire flew from my knee to the rail." The injuries above described were directly due to the passage of the current from the leg to the rail. The chief difficulty experienced in this case was in forming an adequate idea of the extent of the injury and its probable outcome. One of the singular features of the case, and one which I would not *a priori* believe, is that a current of sufficient intensity to produce such extensive local injury could pass through the body without causing instant death. I think a report of a case was given in the *British Medical Journal* some three years ago, of a similar "grounding" of a current which caused instant death, and that the local injury in that case amounted to only a slight charring of the outer aspect of the little finger.

434 West Adams Street.

